# Bangla Speech Emotion Recognition Using Deep Learning-Based Ensemble Learning and Feature Fusion

**DOI:** 10.3390/jimaging11080273

**Published:** 2025-08-14

**Authors:** Md. Shahid Ahammed Shakil, Fahmid Al Farid, Nitun Kumar Podder, S. M. Hasan Sazzad Iqbal, Abu Saleh Musa Miah, Md Abdur Rahim, Hezerul Abdul Karim

**Affiliations:** 1 Department of Computer Science and Engineering, Pabna University of Science and Technology, Pabna 6600, Bangladesh; shakil@vu.edu.bd (M.S.A.S.); nitun@pust.ac.bd (N.K.P.); sazzad@pust.ac.bd (S.M.H.S.I.); 2 Centre for Image and Vision Computing (CIVC), COE for Artificial Intelligence, Faculty of Artificial Intelligence and Engineering (FAIE), Multimedia University, Cyberjaya 63100, Selangor, Malaysia; 3 Department of Computer Science and Engineering, Bangladesh Army University of Science and Technology (BAUST), Saidpur 5311, Bangladesh

**Keywords:** speech-based emotion recognition (SER), data augmentation, feature extraction, MFCC, chromagram features, time–frequency domain feature, visualizable audio representations, ensemble learning, deep learning, LSTM, CNN, handcrafted feature, feature fusion

## Abstract

Emotion recognition in speech is essential for enhancing human–computer interaction (HCI) systems. Despite progress in Bangla speech emotion recognition, challenges remain, including low accuracy, speaker dependency, and poor generalization across emotional expressions. Previous approaches often rely on traditional machine learning or basic deep learning models, struggling with robustness and accuracy in noisy or varied data. In this study, we propose a novel multi-stream deep learning feature fusion approach for Bangla speech emotion recognition, addressing the limitations of existing methods. Our approach begins with various data augmentation techniques applied to the training dataset, enhancing the model’s robustness and generalization. We then extract a comprehensive set of handcrafted features, including Zero-Crossing Rate (ZCR), chromagram, spectral centroid, spectral roll-off, spectral contrast, spectral flatness, Mel-Frequency Cepstral Coefficients (MFCCs), Root Mean Square (RMS) energy, and Mel-spectrogram. Although these features are used as 1D numerical vectors, some of them are computed from time–frequency representations (e.g., chromagram, Mel-spectrogram) that can themselves be depicted as images, which is conceptually close to imaging-based analysis. These features capture key characteristics of the speech signal, providing valuable insights into the emotional content. Sequentially, we utilize a multi-stream deep learning architecture to automatically learn complex, hierarchical representations of the speech signal. This architecture consists of three distinct streams: the first stream uses 1D convolutional neural networks (1D CNNs), the second integrates 1D CNN with Long Short-Term Memory (LSTM), and the third combines 1D CNNs with bidirectional LSTM (Bi-LSTM). These models capture intricate emotional nuances that handcrafted features alone may not fully represent. For each of these models, we generate predicted scores and then employ ensemble learning with a soft voting technique to produce the final prediction. This fusion of handcrafted features, deep learning-derived features, and ensemble voting enhances the accuracy and robustness of emotion identification across multiple datasets. Our method demonstrates the effectiveness of combining various learning models to improve emotion recognition in Bangla speech, providing a more comprehensive solution compared with existing methods. We utilize three primary datasets—SUBESCO, BanglaSER, and a merged version of both—as well as two external datasets, RAVDESS and EMODB, to assess the performance of our models. Our method achieves impressive results with accuracies of 92.90%, 85.20%, 90.63%, 67.71%, and 69.25% for the SUBESCO, BanglaSER, merged SUBESCO and BanglaSER, RAVDESS, and EMODB datasets, respectively. These results demonstrate the effectiveness of combining handcrafted features with deep learning-based features through ensemble learning for robust emotion recognition in Bangla speech.

## 1. Introduction

Speech-based emotion recognition (SER) has applications in natural language processing and human–computer interaction (HCI), among other areas [1]. It can contribute to the enhancement of HCI systems by enabling personalized and human-like interactions. SER is helpful in a number of areas, including marketing, education, mental health, speech synthesis, and customer satisfaction [1]. For instance, SER can enhance the user experience overall by recognizing disgruntled consumers and offering insights into user preferences and behavior. To evaluate speech emotions, a variety of methods and strategies are used, including machine learning algorithms and statistical and probabilistic models [2]. Deep learning techniques have recently been central in this field [2,3,4]. Speech emotion identification has shown promise for deep learning approaches, including CNNs [5], DBNs, RNNs, and LSTMs [6]. Again, not much study or development has been conducted in the Bangla language to identify emotions. Therefore, there is a need and potential to create a speech-emotion recognition system for Bangla. Our study’s main objective is to classify speech emotions using an ensemble learning approach that incorporates three types of deep learning algorithms: CNN, LSTM, BiLSTM, or combinations. Identifying emotions in spoken language, particularly in Bangla, is difficult because of varying linguistic usage, social and cultural factors, subjective experiences, and scant evidence [7,8]. Once more, individual and cultural variations, together with the variety of emotional displays in tones, dialects, and speech rates, present major challenges to algorithms trying to identify emotions in Bangla speech [7,8]. Recently, many researchers employed traditional handcrafted feature-based machine learning approaches such as MFCCs, Chroma, and Spectral Contrast to recognize emotions from Bangla speech [6,9]. However, their performance accuracy is not satisfactory. More recently, some researchers are employing deep learning approaches like CNN, LSTM, and BiLSTM models to improve the performance accuracy [6,10]. However, the mentioned research work still faces challenges in achieving good performance accuracy and generalization properties due to a lack of effective features. To overcome the challenges, we proposed integrating the hand-created and multi-stream deep learning features to develop a Bangla speech-based emotion recognition system. Major contributions of the proposed model are given below:

1. Fusion of Handcrafted and Deep Learning Features: We combine handcrafted features with deep learning-derived representations to capture both explicit speech characteristics and complex emotional patterns. This fusion enhances the model’s accuracy and robustness, improving generalization across different emotional expressions and speaker variations.
2. Handcrafted Features: We extract features such as Zero-Crossing Rate (ZCR), Mel-Frequency Cepstral Coefficients (MFCCs), spectral contrast, and Mel-spectrogram, which focus on key speech characteristics like pitch, tone, and energy fluctuations. Although our approach accepts 1D feature vectors as inputs to the model, some features such as MFCCs, Mel-spectrograms, and chromagrams are themselves generated from time–frequency representations of speech signals that can also be represented as images. This makes our approach sing with imaging-based pipelines of classification that process visual patterns as extracted from audio signals, putting it on the same conceptual level as image-based signal processing. These features provide valuable insights into emotional content, enhancing the model’s ability to distinguish subtle emotional variations.
3. Multi-Stream Deep Learning Architecture: Our model employs three streams: 1D CNNs, 1D CNNs with Long Short-Term Memory (LSTM), and 1D CNNs with bidirectional LSTM (Bi-LSTM), which capture both local and global patterns in speech, providing a robust understanding of emotional nuances. The LSTM and Bi-LSTM streams improve the model’s ability to recognize emotions in speech sequences.
4. Ensemble Learning with Soft Voting: We combine predictions from the three streams using an ensemble learning technique with soft voting, improving emotion classification by leveraging the strengths of each model.
5. Improved Performance and Generalization: Data augmentation techniques such as noise addition, pitch modification, and time stretching enhance the model’s robustness and generalization, addressing challenges like speaker dependency and variability in emotional expressions. Our approach achieves impressive performance, with accuracies of 92.90%, 85.20%, 90.63%, 67.71%, and 69.25% for the SUBESCO, BanglaSER, merged SUBESCO and BanglaSER, RAVDESS, and EMODB datasets, respectively, demonstrating its superiority over traditional models.

## 2. Related Works

Speech emotion recognition (SER) has been transformed by deep learning algorithms [11,12,13]; yet, research on SER in the Bangla language is scarce. A deep learning approach to speech emotion recognition was proposed in 2021 by Sadia Sultana, M. Zafar Iqbal, et al. [14]. Using the Bangla audio-only dataset SUBESCO, they used bidirectional LSTM networks and deep convolutional neural networks with a time-distributed flatten layer for their investigation. They used cross-lingual and multilingual training and testing sets in many tests, and their method with a TDF layer performed better than other cutting-edge CNN-based SER models. In order to conduct cross-lingual experiments in Bangla and English, they employed transfer learning and the SUBESCO and RAVDESS datasets for both cross- and multi-corpus training. The model achieved 86.9% (WA) for the SUBESCO dataset and 82.7% (WA) for the RAVDESS dataset. In 2018, Rahman, Md. Masudur, and associates presented a proposal for an automated voice recognition system for Bengali that included a support vector machine with dynamic time warping [15]. For the static features, they employed MFCCs, and for the dynamic features, MFCC derivatives. SVM with RBF was utilized for classification, and their modified DTW technique was utilized for feature matching. The system achieved 86.08% accuracy on 12 speakers. Using phase-based cepstral features, Chakraborty et al. suggested an automatic speech emotion recognition model in 2022 [8]. They used pre-processing methods with PBCC to extract phase-based information from speech data. The SUBESCO and BanglaSER datasets, which included a gradient-boosting machine-based classifier, were utilized to evaluate the model. In comparison with earlier methods, the findings demonstrated enhanced performance, with an average accuracy of 96% for both speaker-dependent and speaker-independent emotion detection tests. Using five characteristics taken from sound data and used as 1D CNN inputs, Dias Issa, M. Fatih Demirci, et al. created a unique approach for speech emotion identification in 2020 [16]. During testing on the RAVDESS, EMO-DB, and IEMOCAP databases, the model achieved good classification accuracy in speaker-independent audio. For instance, the model achieved 71.61% accuracy on the RAVDESS dataset with eight classes, 86.1% accuracy on EMO-DB (535 samples) in 7 classes, 95.71% accuracy on EMO-DB (520 samples) in 7 classes, and 64.3% accuracy on IEMOCAP in 4 classes. Without the requirement for visual aids, the suggested model performed better than the majority of existing models. Using 2D and 1D CNN LSTM networks, Jianfeng Zhao, Xia Mao, et al. (2018) presented a deep learning method for speech emotion identification [17]. From speech and log-mel spectrograms, their algorithms retrieved features associated with both local and global emotions. They achieved recognition accuracy of 95.33% and 95.89% in speaker-dependent and speaker-independent trials on EmoDB and 52.14% and 89.16% in speaker-independent and speaker-dependent testing on the IEMOCAP database, respectively, outperforming competing methods such as CNN and Deep Belief Network. A 1D dilated CNN with hierarchical features learner blocks (HFLBs) and a bi-directional gated recurrent unit (BiGRU) for SER was suggested by Mustaqeem and Soonil Kwon in 2021 [18]. Through a layered 1D dilated network (HFLBs), they employed spectral analysis to uncover unknown patterns from audio samples. After that, the characteristics were input into the BiGRU, which used a softmax layer to build the likelihood of speech emotions and learn temporal cues. On the IEMOCAP, EMO-DB, and RAVDESS datasets, their model yielded relative accuracy values of 72.75%, 91.14%, and 78.01%, respectively. In 2017, Badshah et al. proposed a model (CNN) with three convolutional and three FC layers [19]. The model was trained using the seven emotion classes found in the EMODB corpus. In order to train, they used spectrograms. The accuracy percentage of their study was 56%. In 2018, Etienne et al. developed the CNN-LSTM model for identifying speech emotions [20]. High-level features were extracted using convolutional layers, while long-term associations were gathered using recurrent layers. Their best-performing model, a convolutional (4 layers) and BLSTM combination (1 layer), produced 61.7% unweighted accuracy and 64.5% weighted accuracy for four emotions.

In 2020, Xusheng Ai, Victor S. Sheng, et al. proposed an ensemble learning approach for speech emotion identification using ACRNN [21]. Convolutional recurrent neural networks were utilized in conjunction with bagging and attention models to handle observation overlap problems. In order to solve further issues, they also employed redagging and augmentation strategies. Their research made use of the Emo-DB and IEMOCAP databases. In 2020, Mustaqeem and Kwon presented an SER model based on spectrogram features that classified emotions using a CNN with deep strides (DSCNN) [22]. The average accuracy for the RAVDESS dataset was 79.5%, whereas the average accuracy for the IEMOCAP dataset was 81.75%. In 2015, Zheng et al. used log-spectrograms as input for their proposed DCNN model for speech emotion recognition [23]. They discovered that their model performed better than conventional models that depended on manually created features after using PCA to minimize dimensionality. In 2021, an ensemble-based cross-lingual speech emotion recognition (SER) approach was proposed by Wisha Zehra et al. [24]. They focused on improving emotion recognition in robots interacting with speakers from multilingual and multicultural backgrounds. Unlike traditional SER systems that use the same corpus for both training and testing, their study trained classifiers on one corpus and tested them on another to simulate real-world multilingual environments. They applied an ensemble learning strategy using a majority voting technique, combining the predictions of multiple classifiers to enhance robustness across corpora. Experiments on Urdu, German, Italian, and English datasets showed improvements in accuracy by up to 13% in within-corpus tests and up to 15% in cross-corpus settings, outperforming several individual classifiers. This demonstrated the advantage of ensemble methods in multilingual SER tasks. A real-time, non-line-of-sight (NLOS) human–machine interaction framework for ensemble-based emotional communication was introduced in 2025 by Shaik Abdul Khalandar Basha, P. M. Durai Raj Vincent, et al. [25]. The authors introduced the EAS framework, which enables long-distance emotional realization through communication. Their system employs CNN-LSTM architectures with and without attention mechanisms for emotional classification and feature extraction. They also used Deep Canonical Correlation Analysis (DCCA) for feature correlation analysis. The CNN-LSTM model with attention design improved in classification accuracy to 87.08%, outperforming baseline CNN (81.11%) and LSTM (84.01%) models. The approach performed better than expected in real-world emotional communication scenarios, which proves the benefit of ensemble and attention-oriented solutions in real-world SER usage.

Although our research focused on emotion recognition from audio speech, researchers also used other modalities for recognition, including facial expressions, physiological signals such as EEG, body gestures, textual data, and video [26]. These modalities can provide rich biometric cues for emotion detection. While working with a biometric modality such as speech or body gestures, we can extract multiple features and combine them for better performance. Features from different biometric modalities can also be fused for more accurate emotion recognition. In 2023, Baole Fu et al. [27] introduced a novel multimodal emotion recognition approach in which a Multimodal Feature Fusion Neural Network (MFFNN) was employed to fuse EEG and eye movement signals. The model consists of a dual-branch feature extraction module and a multi-scale fusion mechanism with cross-channel soft attention to extract complementary spatial and temporal information. On four-class emotion classes of the SEED-IV dataset, the model was proven to attain an accuracy rate of 87.32%, demonstrating the effectiveness of feature fusion in improving emotion recognition performance.

## 3. Datasets

By using the SUBESCO and BanglaSER datasets, which are collections of Bengali speech samples with annotations of expressed emotions, our work seeks to develop an SER system for the language. The RAVDESS and EMODB datasets, each with seven emotion categories, were also used to assess our models. We conducted experiments using five or seven emotion classes, depending on the available labels in each dataset. For example, SUBESCO has seven emotion classes: neutral, angry, disgust, happy, sad, surprise, and fear. We did not select the emotion categories independently; rather, they are predefined within the datasets based on their original annotation protocol.

### 3.1. SUBESCO Dataset

SUBESCO, also known as the SUST Bangla Emotional Speech Corpus, is an audio-only dataset including about seven hours of emotional Bangla speech. It is made up of 7000 utterances that were recorded by 20 native speakers who are split equally between the sexes. Each speaker recorded 10 lines, which correspond to seven different moods. Each audio clip underwent four rounds of verification by male and female raters after the dataset was examined by fifty college students [28]. Table 1 shows emotion classes in the SUBESCO dataset with counts and percentages. It was developed to help in the development of Bangla SER systems and is publicly available.

### 3.2. BanglaSER Dataset

BanglaSER is a collection of speech-audio data from 34 speakers that reflect five fundamental emotional states, and it is used for SER in the Bangla language. Using computers and cell phones to capture spoken audio, 1467 recordings in total of three phrases per recording were made, with an equal number of recordings in each category and an equal distribution of male and female performers (17 male, 17 female) [29]. Table 2 shows emotion classes in the BanglaSER dataset with counts and percentages.

### 3.3. SUBESCO and BanglaSER Merged Dataset

We conducted an inquiry to develop a multi-corpus classification model for Bangla SER by merging the SUBESCO and BanglaSER datasets. Making a more broadly applicable SER model was the primary goal. BanglaSER offers five different emotion courses: fear, neutral, sad, disgusted, and pleased. SUBESCO offers seven different emotional lessons. Table 3 shows emotion classes in the merged dataset with counts and percentages.

### 3.4. RAVDESS Dataset

The RAVDESS collection is made up of audio and video clips of actors performing heartfelt songs and speeches. It is used for research and development in speech and audio processing, multimedia content analysis, and emotional analysis. It contains recordings of seven emotions sung by 24 performers. Three different forms of data are included in the dataset: audio-only, audio–video, and video-only [30]. We used audio-only data in our study. Table 4 shows emotion classes in the RAVDESS dataset with counts and percentages.

### 3.5. EMODB Dataset

The Technical University of Berlin developed the free-to-use German emotional database EMODB, which has 535 statements expressing seven different emotions (angry, bored, anxious, fear, happy, disgust, sadness, and neutral) from 10 expert speakers [31]. Table 5 shows emotion classes in the RAVDESS EMODB with counts and percentages.

## 4. Materials and Methods

**Figure 1:**
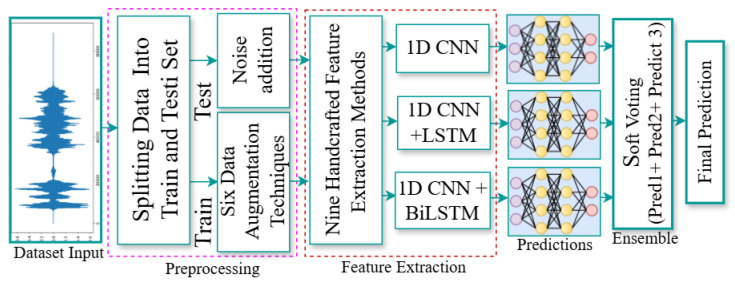
An overview of our proposed methodology.

We propose a novel multi-stream deep learning feature fusion approach for Bangla speech emotion recognition, addressing the limitations of existing methods, such as low accuracy, speaker dependency, and poor generalization across emotional expressions. Our approach begins with various data augmentation techniques applied to the training dataset, improving the model’s robustness and generalization across different speakers and emotional expressions. We extract a comprehensive set of handcrafted features, including ZCR, chromagram, spectral centroid, spectral roll-off, spectral contrast, spectral flatness, MFCCs, RMS energy, and Mel-spectrogram. These handcrafted features capture essential characteristics of the speech signal, providing valuable insights into the emotional content, particularly in terms of pitch, tone, and energy variations. Subsequently, we utilize a multi-stream deep learning architecture to automatically learn complex, hierarchical representations of emotional content in speech. Despite the fact that 2D spectrograms are frequently employed in speech emotion recognition, we concentrated on 1D feature vectors for modeling in this work. The 1D feature vector that we obtained after extracting the aforementioned characteristics naturally fits 1D models such as CNN, LSTM, and BiLSTM. Once more, we wanted to increase efficiency by combining different handcrafted features; in this way, some feature vectors would not need to be converted into 2D form while using 1D input. So, our architecture consists of three distinct streams: the first stream employs 1D CNN, the second integrates 1D CNN with LSTM, and the third stream combines 1D CNN with Bi-LSTM. These deep learning models capture intricate emotional nuances, offering a deeper understanding of speech emotion than handcrafted features alone. We fuse both handcrafted and deep learning-derived features, combining traditional signal characteristics with learned representations of emotional content. We combine predictions from the three streams using an ensemble learning technique with soft voting, improving emotion classification by leveraging the strengths of each model. We assess our model using three primary datasets—SUBESCO, BanglaSER, and a merged version of both—as well as two external datasets, RAVDESS and EMODB. An overview of the proposed methodology is provided in Figure 1.

### 4.1. Data Augmentation

**Figure 2:**
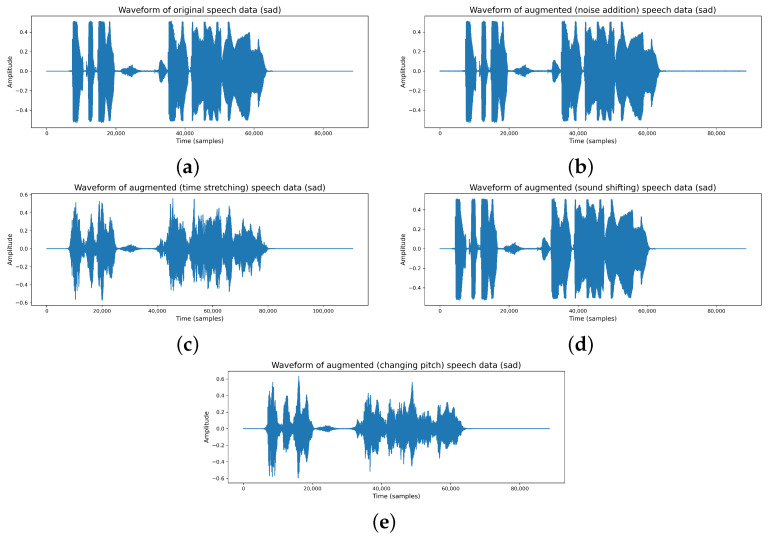
Examples of data augmentations: (**a**) Original. (**b**) Noise Added. (**c**) Time Stretched. (**d**) Sound Shifted. (**e**) Pitch Changed.

To assess the efficacy of a speech-emotion classification algorithm, the dataset is typically split into training and testing sets. In this study, we split the dataset into training and testing sets in the ratios of 70/30 and 80/20 based on the dataset owner protocol. An overview of the train–test samples is provided in Table 6. Then we applied a data augmentation approach on the training dataset, which is described in Table 7. We used a data augmentation technique to produce fresh synthetic training samples by making small adjustments to the original training dataset [9,32]. In this case, we applied six different audio data augmentations to improve the speech emotion detection models. It is important to bear in mind that data augmentation needs to be limited to the training data once it has been split into train and test samples. On our training data, we applied polarity inversion, noise addition, time stretching, pitch change, sound shifting, and random gain [33,34,35]. To expand the number of test samples, we only added noise to our test data. Some examples of data augmentations are given in Figure 2.

### 4.2. Feature Extraction

**Figure 3:**
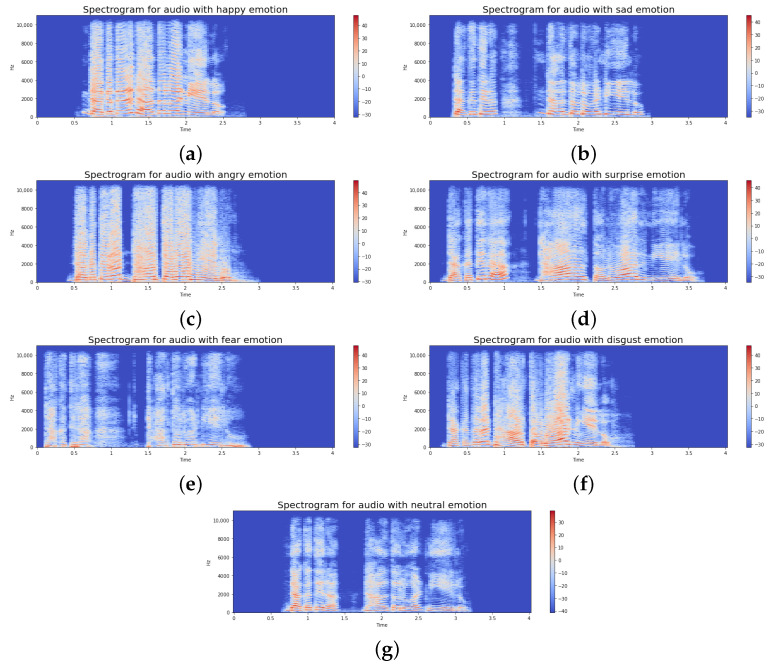
Spectrograms of audio signals from different emotional categories: (**a**) Happy. (**b**) Sad. (**c**) Angry. (**d**) Surprised. (**e**) Fearful. (**f**) Disgusted. (**g**) Neutral.

We extracted nine audio features from the frequency, time, and time–frequency domains, including low- and mid-level features, to categorize emotions in speech. Although our feature extraction process utilizes 1D handcrafted features such as MFCCs, chromagram vectors, and spectral features, they are all derived from time–frequency representations of the speech signal. To visually indicate how emotions manifest in the frequency domain, spectrograms of audio samples from different emotion classes are presented in Figure 3. At first, we loaded the audio files using a fixed duration of 2.5 s with an offset of 0.6 s to avoid silence. We extracted the predefined acoustic features by computing their frame-wise values and then averaging them across all frames to obtain a single fixed-length vector per feature type. The features were concatenated into a single 1D vector using horizontal stacking (np.hstack). These features were extracted for both the original and augmented datasets. All resulting feature vectors are vertically stacked (np.vstack). These final feature vectors are directly used as inputs to the model’s dense layers in a conventional machine learning pipeline. Feature extraction was performed using the Python (v3.10.12) Librosa package (v0.10.1) [33]. The names of the nine features and other relevant information are shown in Table 8.

### 4.3. Deep Learning Model

After the data are prepared, the model has to be trained for classification. This is the process of giving the algorithm a large number of labeled samples to increase its accuracy. A loss function is used to minimize the discrepancy between the predicted and actual outputs. We were able to train three different models: a 1D CNN, a 1D CNN-LSTM, and a 1D CNN-bidirectional LSTM.

#### 4.3.1. 1D-CNN Approach

**Figure 4:**
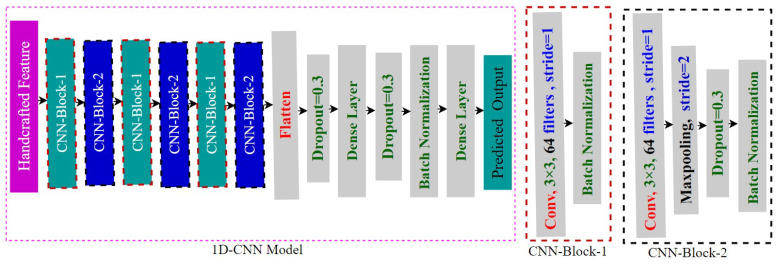
Architecture of our 1D CNN model.

First, we use a 1D-CNN, which has the same architecture across all five datasets, as our emotion classification model. The model includes several layers for one-dimensional convolution, pooling, batch normalization, dropout, activation, flattening, and fully connected layers [48,49]. The convolution layers extract deep features and produce feature maps [49]. Max-pooling is used by the pooling layers, which down-sample and optimize the spatial size of the feature maps [49,50]. The model with ReLU incorporates non-linearity through activation layers [49]. While batch normalization layers hasten deep neural network training, dropout layers prevent overfitting [51,52]. One dimension is extracted from the input by the Flatten layer [53]. The fully connected layer uses a Softmax function to create a probability distribution across the classes to produce predictions based on data from previous layers [49,54]. With a learning rate of 0.00001, Adam was employed as the optimizer [55]. Our proposed 1D CNN model comprises six convolutional layers (filters: 64–256, kernel sizes: 3 and 5), each followed by ReLU activation, batch normalization, and max pooling (pool sizes: 3 or 5, stride: 2). Dropout (rate: 0.3) is applied to reduce overfitting. The fully connected layer has 1024 units, followed by a softmax output layer (5 or 7 units depending on the dataset). The model is trained using a batch size of 32 for up to 100 epochs. The architecture of our 1D CNN model is given in Figure 4.

#### 4.3.2. Integration of 1D-CNN and LSTM Approach

**Figure 5:**
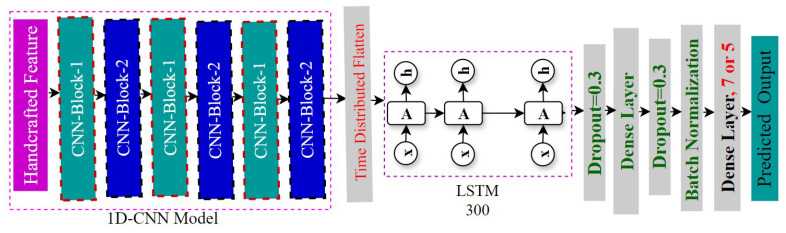
Architecture of our 1D CNN LSTM model.

For our second classification model, we merged an LSTM with a 1D CNN in an attempt to use both models’ advantages. We use a CNN component for feature extraction, where the CNN structure remains unchanged from our 1D CNN model, and the model’s architecture is the same for all five types of datasets. After processing the CNN architecture’s output, a time-distributed flatten layer sends it to an LSTM layer for sequential analysis and a fully connected layer for predictions. The LSTM layer has 300 units, and dropout (rate: 0.3) is applied after the LSTM and dense layers. To make sure that the same weights and biases are given to each temporal timestep of the layer, a wrapper called the time-distributed flatten method was used [14]. A Softmax function is used for probability distribution over emotion categories [54]. The Adam optimizer was used with a 0.000001 learning rate [55]. The model is trained using a batch size of 32 for up to 100 epochs. An architecture of our 1D CNN-LSTM model is given in Figure 5.

#### 4.3.3. Integration of 1D-CNN and Bi-LSTM Approach

**Figure 6:**
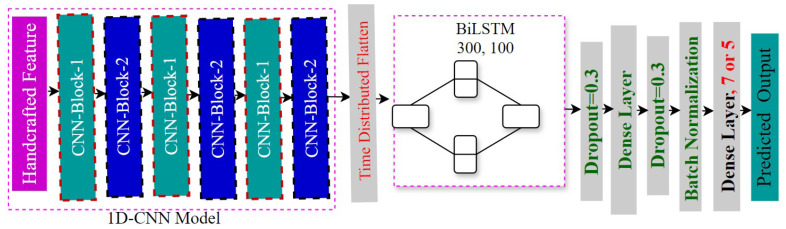
Architecture of our 1D CNN BiLSTM model.

Our third classification model extracts contextual and deep characteristics from the input data by combining two bidirectional LSTM layers (300 and 100 units) with a 1D CNN (same architecture as previous models). Similar to our second model, the CNN output is sent to the LSTM layers via a time-distributed flatten layer; however, this time, the layers are bidirectional, allowing for the acquisition of information from both past and future data points [14]. The input is categorized into one of seven or five emotion categories by the model using a Softmax layer [54]. Utilizing a 0.000001 learning rate, the Adam optimizer was used [55]. The dense layer has 1024 units. It also uses a batch size of 32 and is trained for up to 100 epochs. The architecture of our 1D CNN-BiLSTM model is given in Figure 6.

### 4.4. Soft Voting Ensemble Learning

We trained three classification models and then applied an ensemble learning technique. Using the sum rule ensemble technique, the three classification models (1D CNN, 1D CNN LSTM, and 1D CNN BiLSTM) trained in the prior stage were combined for better emotion categorization. Sum rule ensemble learning (a form of soft voting) aggregates predictions from various models for each class label and predicts the class label based on the highest summed probability, as shown in Figure 1 [56]. In this approach, the overall accuracy is improved by focusing the models on different data points and combining their predictions. The procedure involves generating predictions from each model and aggregating them to form a final prediction. The sum rule is given by Equation (1):

(1) $${\hat{y}}_{\mathrm{final}}=\arg\max\left(\sum_{i=1}^{n}P\left(y|x,\theta_{i}\right)\right)$$

where ${\hat{y}}_{\mathrm{final}}$ is the predicted class label. $P(y|x,\theta_{i})$ is the probability prediction for class *y* from model *i* for input *x*, with parameters $\theta_{i}$. *n* is the total number of models in the ensemble. The prediction is based on the highest summed probability across all models. After generating these summed predictions, we apply the argmax function to convert continuous values into class labels. Finally, we compare the predicted class labels with the ground truth labels to assess the ensemble’s accuracy. An accuracy metric from scikit-learn, such as accuracy_score, is used for this evaluation.

## 5. Results

Three classification models and an ensemble learning strategy were tested on each of the five datasets that we used. Across the datasets, we saw notable variations in the model’s performance, and our ensemble technique continuously increased accuracy.

### 5.1. Ablation Study

Table 9 displays the ablation study where we show the performance for each of our suggested models. Our investigation revealed that the 1D CNN-LSTM model outperformed other base models on the SUBESCO and BanglaSER datasets. Conversely, the 1D CNN model outperformed the others for the SUBESCO and BanglaSER merged, RAVDESS, and EMODB datasets. Overall, our models performed better on the Bangla speech datasets than the other two. The observed accuracy differences can be attributed to variations in language, dataset size, and recording style. SUBESCO and BanglaSER, both Bengali speech corpora, yielded consistently higher performance, possibly indicating that our model generalizes better within the Bengali language domain. In contrast, RAVDESS (English) and EMODB (German) are acted and linguistically different, which may limit cross-lingual generalization.

### 5.2. Outcomes of the Models for SUBESCO Dataset

For the SUBESCO Bangla Speech Emotion Corpus, our proposed models performed well. Over 99% accuracy was achieved by each model in learning the training set of data. The three 1D CNN classification models, 1D CNN LSTM, 1D CNN BiLSTM, and 1D CNN, have testing accuracy scores of 90.93%, 90.98%, and 90.50%, respectively. Ultimately, the accuracy of the ensemble learning approach that combined the three models was 92.90%. This indicates a significant increase in accuracy. A summary of all these findings is given in Table 10. In Table 11, the accuracy of the Ensemble Learning approach for each emotion class of the SUBESCO dataset is given, and the performance metrics are given in Table 12.

**Figure 7:**
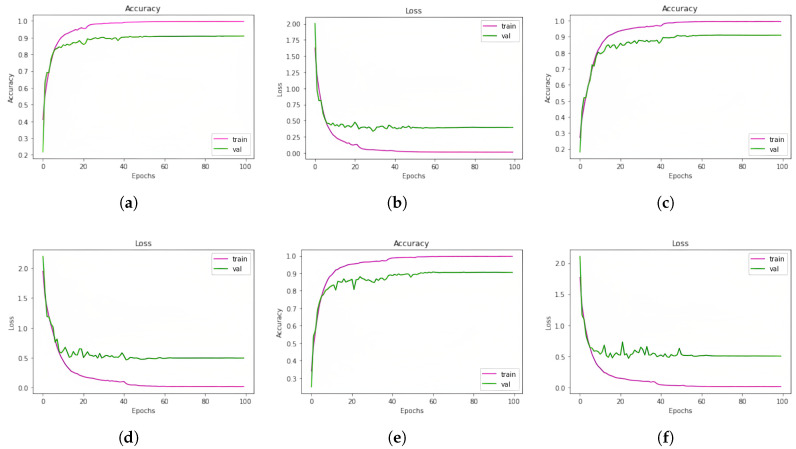
Learning curves (accuracy and loss) of the models (1D CNN, 1D CNN-LSTM, and 1D CNN-BiLSTM, respectively) for the SUBESCO dataset. (**a**) 1D CNN accuracy curve; (**b**) 1D CNN loss curve; (**c**) 1D CNN-LSTM accuracy curve; (**d**) 1D CNN-LSTM loss curve; (**e**) 1D CNN-BiLSTM accuracy curve; (**f**) 1D CNN-BiLSTM loss curve.

In Figure 7, the learning curves (accuracy and loss) of our trained models for the SUBESCO dataset are given.

### 5.3. Outcomes of the Models for BanglaSER Dataset

**Figure 8:**
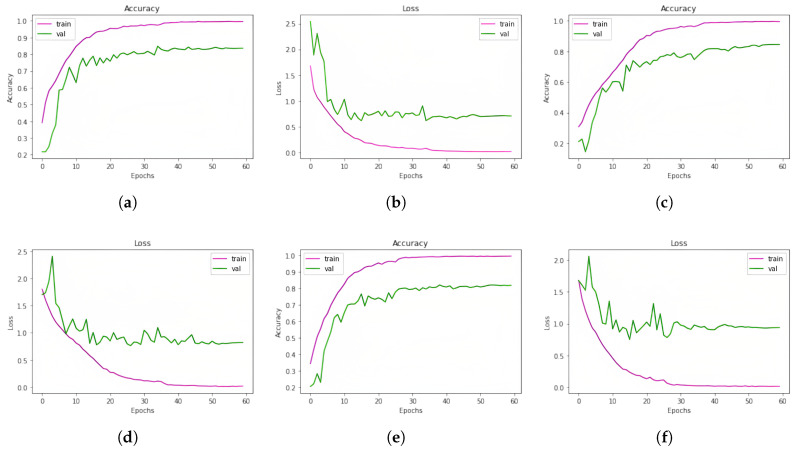
Learning curves (accuracy and loss) of the models (1D CNN, 1D CNN-LSTM, and 1D CNN-BILSTM, respectively) for the BanglaSER dataset. (**a**) 1D CNN accuracy curve; (**b**) 1D CNN loss curve; (**c**) 1D CNN-LSTM accuracy curve; (**d**) 1D CNN-LSTM loss curve; (**e**) 1D CNN-BiLSTM accuracy; (**f**) 1D CNN-BiLSTM loss curve.

On the BanglaSER dataset, the accuracy of the three classification models 1D CNN, 1D CNN LSTM, and 1D CNN BiLSTM was 83.67%, 84.52%, and 81.97%, in that order. Ultimately, the accuracy of the ensemble learning method that integrates the three models was 85.20%. This indicates an improvement in accuracy. A summary of all these findings is given in Table 13. In Table 14, the accuracy of the Ensemble Learning approach for each emotion class of the BanglaSER dataset is given, and the performance metrics are given in Table 15. The learning curves (accuracy and loss) of the models (1D CNN, 1D CNN-LSTM, and 1D CNN-BILSTM, respectively) for the BanglaSER dataset are given in Figure 8.

### 5.4. Outcomes of the Models for SUBESCO and BanglaSER Merged Dataset

**Figure 9:**
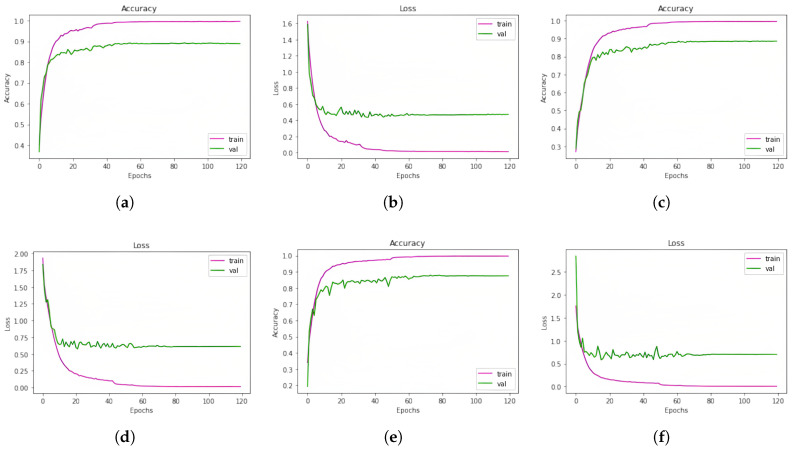
Learning curves (accuracy and loss) of the models (1D CNN, 1D CNN-LSTM, and 1D CNN-BILSTM, respectively) for the SUBESCO and BanglaSER merged dataset. (**a**) 1D CNN accuracy curve; (**b**) 1D CNN loss curve; (**c**) 1D CNN-LSTM accuracy curve; (**d**) 1D CNN-LSTM loss curve; (**e**) 1D CNN-BiLSTM accuracy curve; (**f**) 1D CNN-BiLSTM loss curve.

In order to evaluate the three classification models (1D CNN, 1D CNN LSTM, and 1D CNN BiLSTM), we combined two Bangla speech emotion datasets. On the combined dataset, the models’ accuracy was 88.92%, 88.61%, and 87.56%, in that order. Nonetheless, we obtained an accuracy of 90.63% with an ensemble learning approach that included all three models, suggesting an improvement in accuracy. A summary of model accuracies for the SUBESCO and BanglaSER merged dataset is given in Table 16. In Table 17, the accuracy of the Ensemble Learning approach for each emotion class of the SUBESCO and BanglaSER merged dataset is given, and the performance metrics are given in Table 18. The learning curves (accuracy and loss) of the models for the SUBESCO and BanglaSER merged dataset are given in Figure 9.

**Figure 10:**
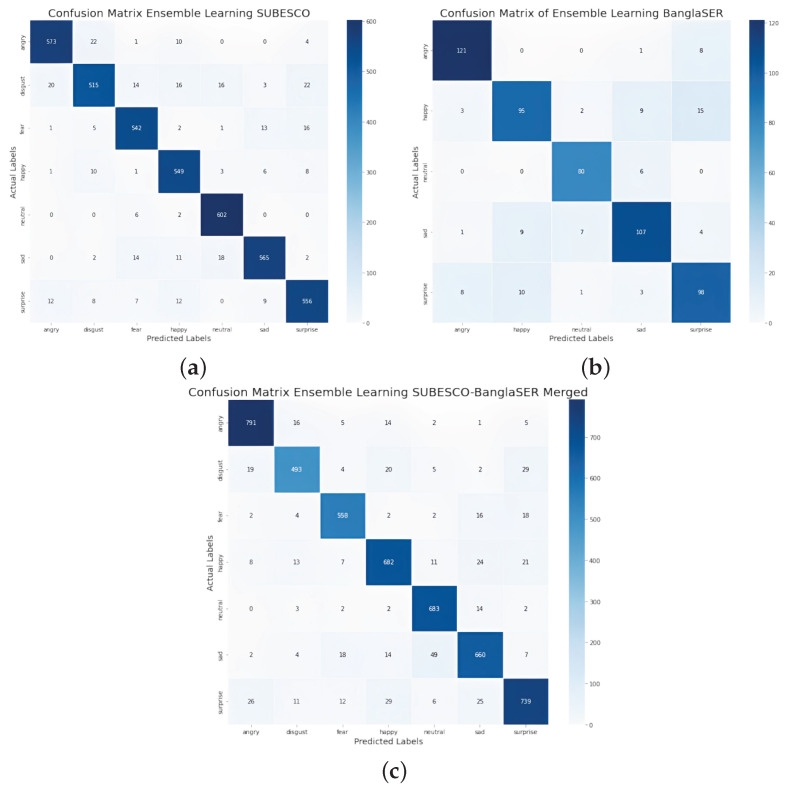
Confusion matrices of the ensemble learning approach for different datasets: (**a**) SUBESCO; (**b**) BanglaSER; (**c**) SUBESCO and BanglaSER merged.

For all of our datasets except EMODB, the ensemble learning approach showed improved classification accuracy. Figure 10 shows the confusion matrices of the ensemble learning approach for the SUBESCO, BanglaSER, and SUBESCO-BanglaSER merged datasets, where each matrix provides a detailed view of the classification outcomes of the ensemble learning approach for each of the datasets. For the SUBESCO dataset, while overall accuracy was high, our model faced difficulty distinguishing between ‘disgust’ and ‘sad,’ and occasionally misclassified ‘surprise’ as ‘angry’ or ‘disgust’; ‘neutral’ expressions, however, were recognized very accurately. For the BanglaSER dataset, we noted good performance for ‘angry,’ ‘neutral’, ‘sad’, and ‘surprise’, but more errors with ‘happy’, which was sometimes confused with ‘surprise’ or ‘sad’, suggesting potential feature overlap in that dataset. Merging the BanglaSER dataset with SUBESCO led to an overall improvement in accuracy and a reduction in the confusion between ‘disgust’ and ‘sad.’ However, there was still some confusion for ‘surprise’. By comparing these matrices, we can evaluate the performance of our ensemble learning approach, which provided improved identification of emotion classes and handled class imbalance and misclassification across all the datasets.

### 5.5. Outcomes of the Models for RAVDESS and EMODB Datasets

The models on the RAVDESS and EMODB datasets were trained using the identical model setups as the previous datasets. The models’ accuracy on these datasets was mediocre; however, our ensemble learning model’s accuracy increased. The accuracy of our models for the RAVDESS and EMODB datasets is given in Table 19 and Table 20, respectively.

### 5.6. State of the Art Comparison

Table 21 presents a comparison of various state-of-the-art models in emotion recognition using speech datasets. Each study employs distinct features and models, with a variety of accuracy metrics for different datasets. For instance, Sultana et al. [14] achieved an accuracy of 86.9% on the SUBESCO dataset using Mel-spectrogram and Deep CNN combined with BLSTM, while Rahman et al. [15] used MFCCs and their derivatives with SVM and DTW, achieving 86.08% accuracy on Bangla 12 speakers. Chakraborty et al. [8] utilized PBCC features with a Gradient Boosting Machine, reaching an impressive 96% accuracy on their dataset. Notably, the proposed model demonstrates superior performance, with 92.90% accuracy on the SUBESCO dataset, and notable results on RAVDESS, EMO-DB, BanglaSER, and a combined dataset (SUBESCO + BanglaSER) with 90.63% accuracy. This highlights the robustness and effectiveness of the proposed ensemble model.

### 5.7. Discussion

Table 9 shows the ablation study, and Table 21 shows the state of the art comparison model. For every dataset except EMODB, the accuracy of the ensemble learning strategy was better than that of other base models. Establishing a method for improved speech emotion categorization from Bangla speech was the main goal of our research. Our method demonstrates how the classification accuracy of Bangla speech may be increased by combining the right features with data augmentations, followed by an ensemble of trained models. Although we did not directly use spectrogram or chromagram images as model inputs, the features we extracted (such as MFCCs and chromagrams) are processed from these representations. Their 2D structure and visualization potential establish a conceptual link with imaging methodologies, even though we process them as 1D feature vectors. In contrast to Sadia Sultana, M. Zafar Iqbal, et al. (2021), who used CNN and the BiLSTM model to obtain 86.9% accuracy on the SUBESCO dataset [14], our method achieved 92.90% accuracy on the SUBESCO dataset, 85.20% accuracy on the BanglaSER dataset, and 90.63% accuracy on the combined dataset of SUBESCO and BanglaSER. Regarding the classification of emotions from speech, the ensemble learning approach performs better than most of the models. One main limitation of our research is that our experiment has been conducted on acted datasets only. Our experiment was limited to the acted dataset, which is one of the key limitations of our study. In real-world situations, our model might not function well because of variances in speech data caused by environments, languages, and cultural differences. It is also important to take into account the various Bangla dialects. In a subsequent study, we may expand to incorporate natural speech data rather than just acted data to more accurately detect speech patterns and emotional expressions in real-world situations. Moreover, speech data from multiple regional dialects of the language or multilingual datasets may be integrated to create a more robust system that can accurately recognize emotions in a range of languages and cultural contexts. We may look into several sets of data augmentation and feature extraction strategies to find out which combinations perform best together for voice emotion recognition. We can expand upon our work by evaluating model robustness under adversarial or environmental audio perturbations. To improve the ensemble learning method, we may eventually try incorporating more categorization models and experimenting with other combinations. Our ensemble learning strategy demonstrated constant superiority over the other models on all datasets. Additionally, our method’s efficacy with Bengali datasets is demonstrated by the results obtained. With good accuracy levels on most datasets, our method demonstrated the overall effectiveness of ensemble learning for speech-emotion recognition.

## 6. Conclusions

In this study, we propose a novel multi-stream deep learning feature fusion approach for Bangla speech emotion recognition, which effectively addresses the challenges of low accuracy, speaker dependency, and poor generalization. By combining handcrafted features with deep learning-derived features and employing an ensemble learning technique, our method significantly enhances the robustness and accuracy of emotion recognition across diverse datasets. We combine predictions from the three streams using an ensemble learning technique with soft voting, improving emotion classification by leveraging the strengths of each model. The results demonstrate the ability to capture intricate emotional nuances in Bangla speech, achieving high performance in both primary and external datasets. This approach holds broader potential for expanding emotion recognition systems to other languages and emotional categories, paving the way for more universal and accurate speech-emotion recognition systems. Looking ahead, we plan to extend this research by applying the proposed approach to other underrepresented languages and exploring additional emotional categories. We aim to enhance the generalization capability of the model by incorporating more diverse datasets and refining the feature extraction techniques. Furthermore, future work will focus on integrating this emotion recognition model into real-time systems, such as virtual assistants and conversational AI platforms, to improve their responsiveness and empathy in human–machine interactions.

## Figures and Tables

**Table 1:** Emotion classes in the SUBESCO dataset with counts and percentages.

Class Name	Total Count	Percentage
Happy	1000	14.29
Sad	1000	14.29
Angry	1000	14.29
Surprise	1000	14.29
Fear	1000	14.29
Disgust	1000	14.29
Neutral	1000	14.29

**Table 2:** Emotion classes in the BanglaSER dataset with counts and percentages.

Class Name	Total Count	Percentage
Happy	306	20.87
Sad	306	20.87
Angry	306	20.87
Surprise	306	20.87
Neutral	243	16.53

**Table 3:** Emotion classes in the merged SUBESCO and BanglaSER dataset.

Class Name	Total Count	Percentage
Happy	1306	15.41
Sad	1306	15.41
Angry	1306	15.41
Surprise	1306	15.41
Fear	1000	11.80
Disgust	1000	11.80
Neutral	1243	14.67

**Table 4:** Emotion classes in the RAVDESS dataset.

Class Name	Total Count	Percentage
Happy	192	13.33
Sad	192	13.33
Angry	192	13.33
Surprise	192	13.33
Fear	192	13.33
Disgust	192	13.33
Neutral	96	6.67

**Table 5:** Emotion classes in the EMODB dataset.

Class Name	Total Count	Percentage
Happy	71	13.27
Sad	62	11.59
Angry	127	23.74
Boredom	81	15.14
Fear	69	12.90
Disgust	46	8.60
Neutral	79	14.77

**Table 6:** Overview of our train–test samples.

Dataset Name	Total Samples	Train/Test Ratio	Train Samples	Test Samples
SUBESCO	7000	70/30	4900	2100
BanglaSER	1467	80/20	1173	294
SUBESCO and BanglaSER merged	8467	70/30	5926	2541
RAVDESS (Audio-only)	1248	70/30	1008	432
EMODB	535	80/20	428	107

**Table 7:** Data augmentation techniques and their descriptions.

Augmentation Name	Description
**Polarity Inversion**	Reverses the phase of the audio signal by multiplying it by −1, effectively canceling the phase when combined with the original signal, resulting in silence [36]. It can simulate variations in recordings caused by microphone polarity errors, which may slightly enhance the generalization of the model. Polarity inversion was applied by multiplying the signal by −1.
**Noise Addition**	Adds random white noise to the audio data to enhance its variability and robustness [9]. We have added white Gaussian noise at 10 dB and 20 dB SNR using numpy.random.normal() with a noise factor of 0.005.
**Time Stretching**	Alters the speed of the audio by stretching or compressing time series data, increasing or decreasing sound speed [9]. We have applied time stretching on our audio data with a rate (stretch factor) of 0.8 using librosa.time_stretch() function.
**Pitch Change**	Changes the pitch of the audio signal by adjusting the frequency of sound components, typically by resampling [37]. We have shifted ±2 semitones of our audio data with a pitch factor (steps to shift) of 0.8 using librosa.effects.pitch_shift() function.
**Sound Shifting**	Randomly shifts the audio by a predefined number of seconds, introducing silence at the shifted location if necessary [9]. We have shifted audio ±5 ms using numpy.roll() with shifts from random.uniform(−5, 5).
**Random Gain**	Alters the loudness of the audio signal using a volume factor, making it louder or softer [38]. To apply random gain, we randomly adjusted volume between ×2 and ×4 using random.uniform().

**Table 8:** Feature extraction and their advantages.

Feature Name	Description and Advantage
**Zero-Crossing Rate (ZCR)**	Counts the number of times the audio signal crosses the horizontal axis. It helps analyze signal smoothness and is effective for distinguishing voiced from unvoiced speech [39,40].
**Chromagram**	Represents energy distribution over frequency bands corresponding to pitch classes in music. It captures harmonic and melodic features of the signal, useful for tonal analysis [41].
**Spectral Centroid**	Indicates the “center of mass” of a sound’s frequencies, providing insights into the brightness of the sound. It is useful for identifying timbral characteristics [42].
**Spectral Roll-off**	Measures the frequency below which a certain percentage of the spectral energy is contained. This feature helps in distinguishing harmonic from non-harmonic content [42].
**Spectral Contrast**	Measures the difference in energy between peaks and valleys in the spectrum, capturing timbral texture and distinguishing between different sound sources [43,44].
**Spectral Flatness**	Quantifies how noise-like a sound is. A high spectral flatness value indicates noise-like sounds, while a low value indicates tonal sounds, useful for identifying the type of sound [45].
**Mel-Frequency Cepstral Coefficients (MFCCs)**	Captures spectral variations in speech, focusing on features most relevant to human hearing. It is widely used in speech recognition and enhances emotion recognition capabilities [42,45].
**Root Mean Square (RMS) Energy**	Measures the loudness of the audio signal, offering insights into the energy of the sound, which is crucial for understanding the emotional intensity [46].
**Mel-Spectrogram**	Converts the frequencies of a spectrogram to the mel scale, representing the energy distribution in a perceptually relevant way, commonly used in speech and audio processing [47].

**Table 9:** Summary of performances (accuracy %) of our proposed models.

Dataset	1D CNN	1D CNN LSTM	1D CNN BiLSTM	Ensemble Learning
SUBESCO	90.93%	90.98%	90.50%	92.90%
BanglaSER	83.67%	84.52%	81.97%	85.20%
SUBESCO + BanglaSER	88.92%	88.61%	87.56%	90.63%
RAVDESS	65.63%	64.93%	60.76%	67.71%
EMODB	69.57%	67.39%	65.84%	69.25%

**Table 10:** Model accuracy on the SUBESCO dataset.

Model	Accuracy
1D CNN	90.93%
1D CNN LSTM	90.98%
1D CNN BiLSTM	90.50%
Ensemble Learning	92.90%

**Table 11:** Accuracy of the Ensemble Learning approach for each emotion class of SUBESCO dataset.

Emotion	Accuracy (%)
Angry	93.93
Disgust	84.98
Fear	93.61
Happy	94.98
Neutral	98.69
Sad	92.32
Surprise	92.05

**Table 12:** Performance metrics (Precision, Recall, and F1-Score) of the Ensemble Learning approach for each emotion class of SUBESCO dataset.

Class	Precision (%)	Recall (%)	F1-Score (%)
Angry	94.00	94.00	94.00
Disgust	92.00	85.00	88.00
Fear	93.00	93.00	93.00
Happy	91.00	95.00	93.00
Neutral	94.00	99.00	96.00
Sad	95.00	92.00	94.00
Surprise	91.00	92.00	92.00
**Macro Average**	93.00	93.00	93.00
**Weighted Average**	93.00	93.00	93.00
**Accuracy (%)** = 93.00			

**Table 13:** Model accuracy on the BanglaSER dataset.

Model	Accuracy
1D CNN	83.67%
1D CNN LSTM	84.52%
1D CNN BiLSTM	81.97%
Ensemble Learning	85.20%

**Table 14:** Accuracy of the Ensemble Learning approach for each emotion class of BanglaSER dataset.

Emotion	Accuracy (%)
Angry	93.07
Happy	76.61
Neutral	93.02
Sad	83.59
Surprise	81.67

**Table 15:** Performance metrics (Precision, Recall, and F1-Score) of the Ensemble Learning approach for each emotion class of BanglaSER dataset.

Class	Precision (%)	Recall (%)	F1-Score (%)
Angry	91.00	93.00	92.00
Happy	83.00	77.00	80.00
Neutral	89.00	93.00	91.00
Sad	85.00	84.00	84.00
Surprise	78.00	82.00	80.00
**Macro Average**	85.00	85.00	85.00
**Weighted Average**	85.00	85.00	85.00
**Accuracy (%)** = 85.00			

**Table 16:** Model accuracy on the SUBESCO and BanglaSER merged dataset.

Model	Accuracy
1D CNN	88.92%
1D CNN LSTM	88.61%
1D CNN BiLSTM	87.56%
Ensemble Learning	90.63%

**Table 17:** Accuracy of the Ensemble Learning approach for each emotion class of SUBESCO and BanglaSER merged dataset.

Emotion	Accuracy (%)
Angry	94.84
Disgust	86.19
Fear	92.69
Happy	89.03
Neutral	96.74
Sad	87.53
Surprise	87.15

**Table 18:** Performance metrics (Precision, Recall, and F1-Score) of the Ensemble Learning approach for each emotion class of SUBESCO and BanglaSER merged dataset.

Class	Precision (%)	Recall (%)	F1-Score (%)
Angry	93.00	95.00	94.00
Disgust	91.00	86.00	88.00
Fear	92.00	93.00	92.00
Happy	89.00	89.00	89.00
Neutral	90.00	97.00	93.00
Sad	89.00	88.00	88.00
Surprise	90.00	87.00	89.00
**Macro Average**	91.00	91.00	91.00
**Weighted Average**	91.00	91.00	91.00
**Accuracy (%)** = 91.00			

**Table 19:** Model accuracy on the RAVDESS dataset.

Model	Accuracy
1D CNN	65.63%
1D CNN LSTM	64.93%
1D CNN BiLSTM	60.76%
Ensemble Learning	67.71%

**Table 20:** Model accuracy on the EMODB dataset.

Model	Accuracy
1D CNN	69.57%
1D CNN LSTM	67.39%
1D CNN BiLSTM	65.84%
Ensemble Learning	69.25%

**Table 21:** State of the art comparison of the proposed model.

Research	Features Used	Model	Accuracy for 5 Datasets (%)				
			SUBESCO (Bangla)	RAVDESS (American English)	EMO-DB	BanglaSER	(SUBESCO + BanglaSER)
Sultana et al. [14]	Mel-spectrogram	Deep CNN and BLSTM	86.9	82.7			
Rahman et al. (2018) [15]	MFCCs, MFCC derivatives	SVM with RBF, DTW	86.08 (Bangla 12 speakers)	-			
Chakraborty et al. (2022) [8]	PBCC	P Gradient Boosting Machine		96			96
Issa, et al. [16]	MFCCs, Mel-spectrogram, Chroma-gram, Spectral contrast feature, Tonnetz representation	1D CNN	64.3% (IEMOCAP, 4 classes)	71.61 (8 classes)	95.71		
Zhao, et al. 2019 [17]	Log mel spectrogram	1D CNN LSTM, 2D CNN LSTM	89.16 (IEMOCAP dependent)	52.14% (IEMOCAP independent)		95.33 (Emo-Db dependent)	95.89% (independent)
Mustaqeem et al. [18]	Spectral analysis	1D Dilated CNN with BiGRU	72.75	78.01	91.14	-	-
Badshah et al. (2017) [19]	Spectrograms	CNN (3 convolutional, 3 FC layers)			56%		
Etienne, et al. (2018) [20]	High-level features, log-mel Spectrogram	CNN-LSTM (4 conv + 1 BLSTM layer)	61.7% (Unweighted), 64.5% (Weighted)				
Proposed	ZCR, chroma-gram, RMS, spectral centroid, spectral roll-off, spectral contrast, spectral flatness, mel spectrogram, and MFCCs	Ensemble of 1D CNN, 1D CNN LSTM, and 1D CNN BiLSTM	92.90 % (SUBESCO),	67.71% (RAVDESS),	69.25% (EMODB)	85.20% (BanglaSER),	90.63% (SUBESCO + BanglaSER)

## Data Availability

No new data were created or analyzed in this study. All data used are publicly available from the sources cited in the manuscript.

## Abbreviations

PBCC	Phase-Based Cepstral Coefficients
DTW	Dynamic Time Warping
RMS	Root Mean Square
MFCCs	Mel-Frequency Cepstral Coefficients
